# Adrenocortical pheochromocytoma diagnosed during pregnancy: a case report

**DOI:** 10.1186/s12884-023-05844-7

**Published:** 2023-07-18

**Authors:** Xiaoteng Qiang, Yin Li, Qing Bai, Jing Huang, Xuemei Ma, Weiwei Wang

**Affiliations:** 1 Dali University, Dali City, Yunnan Province China; 2 The Third People’s Hospital of Yunnan Province (The Second Affiliated Hospital of Dali University, 292 Beijing Road, Kunming City, Yunnan Province China

**Keywords:** Pregnancy, Gestational hypertension, Adrenocortical carcinoma

## Abstract

This paper reports a rare case of adrenocortical carcinoma (ACC) diagnosed during pregnancy presenting with gestational hypertension. Hypertensive disorders in pregnancy should receive enough attention to identify and exclude the possibility of adrenal diseases, thereby making a timely diagnosis and active treatment.

## Background

Gestational hypertension, a common complication of pregnancy, remains one of the leading causes of maternal and perinatal fetal and neonatal mortality, which seriously compromises the health of mothers and infants [1, 2]. The etiology of very few patients is related to adrenal tumors. Adrenocortical carcinoma (ACC) is a rare malignant tumor with an incidence rate of about one in a million [3]. ACC is characterized by strong invasiveness, rapid disease progression, and poor prognosis. A large proportion of ACC patients may present local infiltration or distant metastasis at the time of diagnosis. Further, ACC in pregnancy is rather rare, and there are few reports on ACC in pregnancy.

## Case presentation

**Fig. 1:**
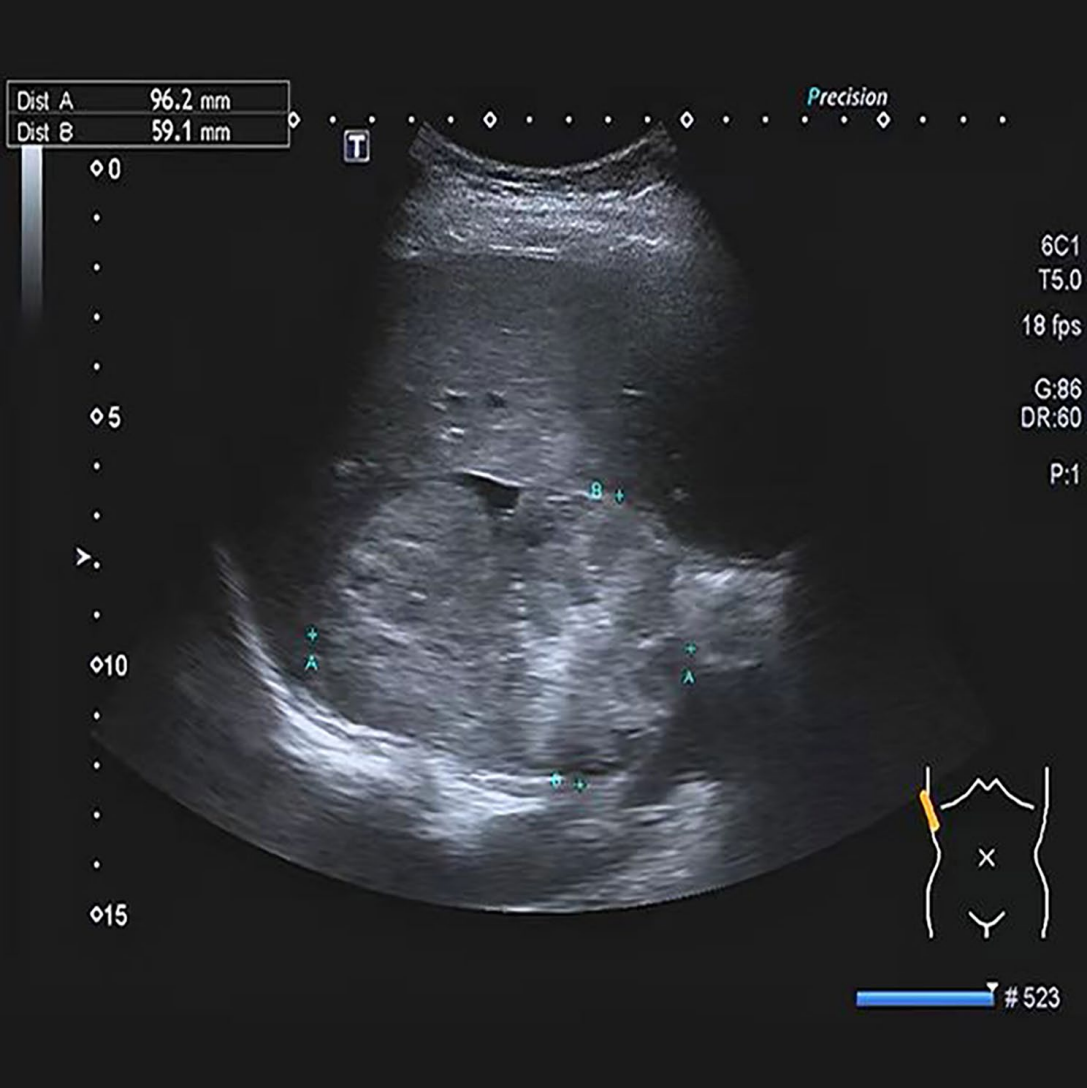
B-ultrasound of the urinary system indicated a cystic and solid space-occupying lesion in the right adrenal gland

**Fig. 2:**
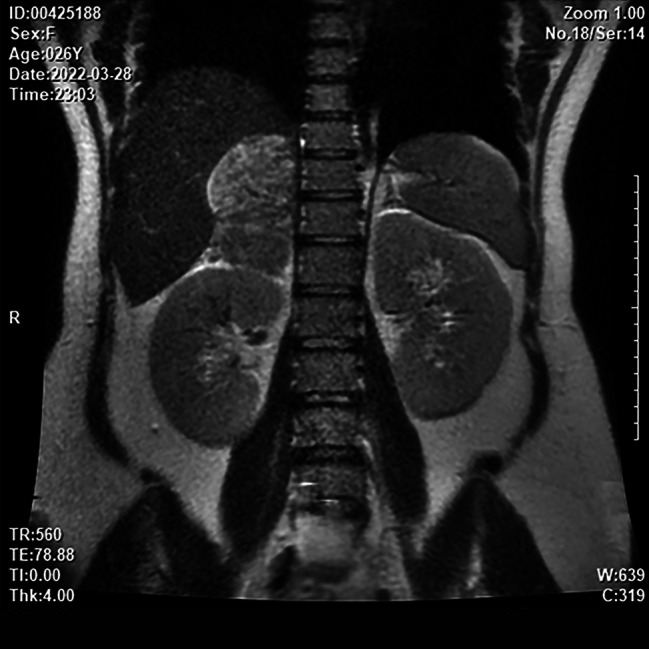
MRI of the urinary system showed a mass in the right adrenal gland with necrosis and hemorrhage

**Fig. 3:**
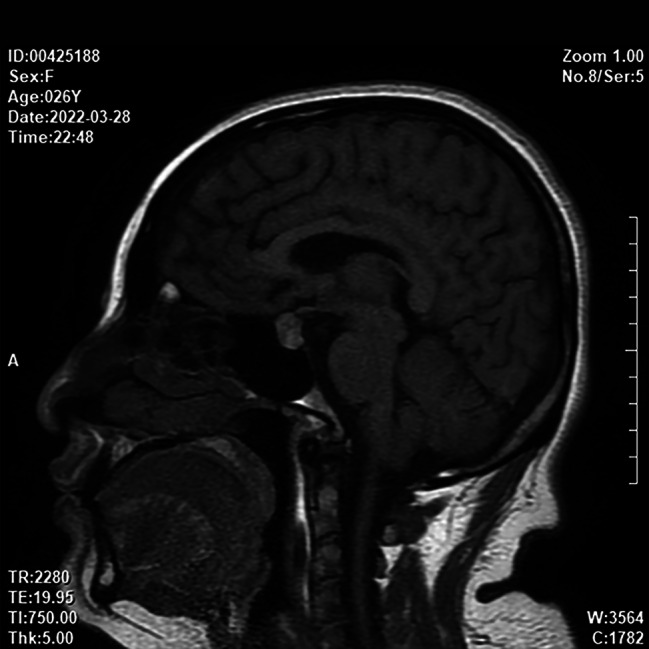
MRI of the brain showed that the pituitary gland was plump

A 26-year-old, gravida 2, para 1 (G2P1) woman at the 31st week of gestation was admitted to the hospital with a chief complaint of elevated blood pressure for 3 months. The woman occasionally suffered from dizziness and amaurosis when changing her posture during pregnancy. Regular antenatal examinations were performed, and there was no sweating or other special discomfort during pregnancy. The oral glucose tolerance test (OGTT) revealed 5.46-8.76-8.42mmol/L, and the patient was diagnosed with gestational diabetes mellitus and given diet and exercise interventions, but no regular blood glucose monitoring. The blood pressure was 106/78 mmHg at the 7th week of gestation, 127/94 mmHg at the 16th week of gestation, and 149/100 mmHg at the 25th week of gestation. However, the patient did not pay attention enough attention to it and also did not receive further diagnosis and treatment. Palpitation and chest tightness occurred once at 31 weeks + 2 days of gestation. The next day, the patient felt headache and dizziness, without blurred vision, and her blood pressure was 161/112 mmHg. Therefore, the patient was transferred to the local hospital. The presence of a solid mass in the right-side adrenal region was identified during the admission obstetric ultrasound evaluation. The Fig. 1 resulted to be highly suggestive of an adrenal pheochromocytoma. The woman presented with severe hypertension (164/109 mmHg), therefore, hydrochloride 100 mg (twice a day, po) was started twice a day. A course of corticosteroids for fetal lung maturation was also prescribed taking into account the high chance of premature delivery (31 weeks + 6 days of gestation). Considering the suspicion of pheochromocytoma and the presence of a severe hypertension the patient was referred to out tertiary obstetric care unit. At 33 weeks + 2 days of gestation, the patient was given detemir insulin hypoglycemic treatment due to poor blood glucose control. After admission, the patient had no dizziness, headache, chest tightness, sweating, abdominal distension, abdominal pain, vaginal bleeding, or other special discomforts. The patient’s vital signs, blood sugar, and consciousness symptoms were monitored, and relevant auxiliary examinations were carried out. The results of the blood biochemical examination are shown in Table 1. For imaging examinations, B-ultrasound of the urinary system indicated a cystic and solid space-occupying lesion in the right adrenal gland, considering the possibility of pheochromocytoma (Fig. 1); The third trimester growth scan has shown a fetus of 2095 g±, with a mild polihydramnios (deepest vertical pocket of amniotic fluid of 10.5 cm); magnetic resonance (MRI) of the urinary system showed a mass in the right adrenal gland with necrosis and hemorrhage, and pheochromocytoma was considered (Fig. 2); MRI of the brain showed that the pituitary gland was plump, and no other obvious abnormality was found (Fig. 3). Multi-disciplinary consultation was conducted to evaluate the patient’s condition comprehensively, and it was recommended to terminate the pregnancy at the 34th week of gestation after her condition gradually stabilized. After maternal hypertension and glucose control was obtained at 34 weeks + 1 day, considering her physical condition and disease state, in order to reduce the risk of continuing pregnancy, the patient and their family required an elective Caesarean section. No complication occurred during surgery and a healthy newborn was delivered. After the operation, the patient continued to receive nifedipine controlled-release tablets 30 mg (one a day, po) and labetalol 150 mg (once every 8 h, po) to control the blood pressure between 128–136/85–93 mmHg and also received infection prevention, blood pressure reduction, and symptomatic treatment. Five days after cesarean section the patient was discharged from the hospital with the stable condition, and she was instructed to continue to take oral nifedipine controlled-release tablets and labetalol to control blood pressure.

**Table 1:** The results of the blood biochemical examination

Biochemical examination			
Item		Measured value	Normal reference value
Plasma cortisol		336.41 ng/ml	30–180 ng/ml
Aldosterone		368.21 pg/ml	10–160 pg/ml
Plasma rennin		53.96 pg/ml	4–22 pg/ml
Angiotensin II		89.70 pg/ml	25–129 pg/ml
Adrenocorticotropic hormone		2.24 pg/ml	7–64 pg/ml
Aldosterone/plasma renin		6.82	< 50
Dopamine		<65.2 pmol/l	< 888 pmol/l
Adrenaline		<55.5 pmol/l	< 480 pmol/l
Noradrenaline		346.8 pmol/l	515–3240 pmol/l
Urinary vanillylmandelic acid		6.3 mg/24H	2–7 mg/24H
24-hour urinary free metanephrines	Metanephrine	48 nmol/24H	< 800 nmol/24H
	Normetanephrine	132 nmol/24H	< 800 nmol/24H
	3-Methoxytyramine	161 nmol/24H	< 800 nmol/24H
Plasma metanephrines	3-Methoxytyramine	<0.08 nmol/l	<6.6 nmol/l
	Metanephrine	<0.08 nmol/l	<6.6 nmol/l
	Normetanephrine	0.18 nmol/l	<6.6 nmol/l
24-hour urinary free cortisol	24-hour urinary free cortisol concentration	154 ng/ml	25–125 ng/ml
	24-hour urinary free cortisol content	492.8 ug/l*24 h	25–125 ug/l*24 h

**Fig. 4:**
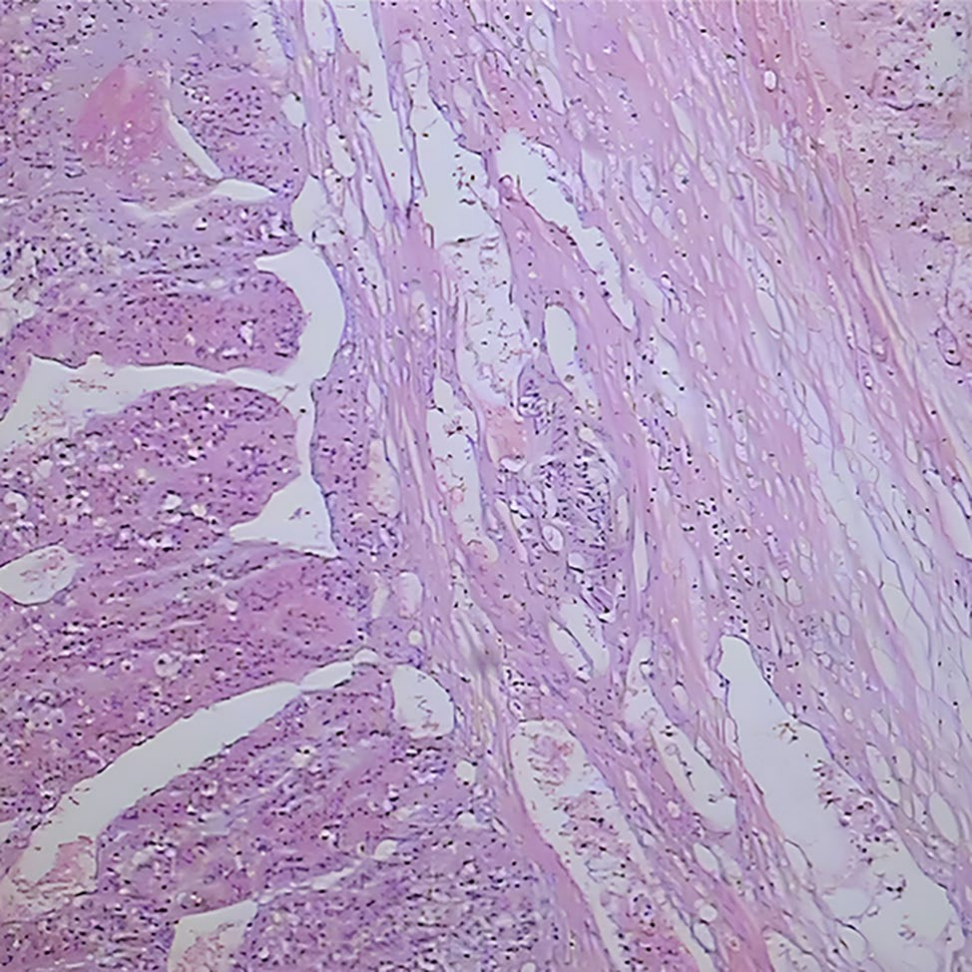
Cancer cells were arranged in an acinar shape

**Fig. 5:**
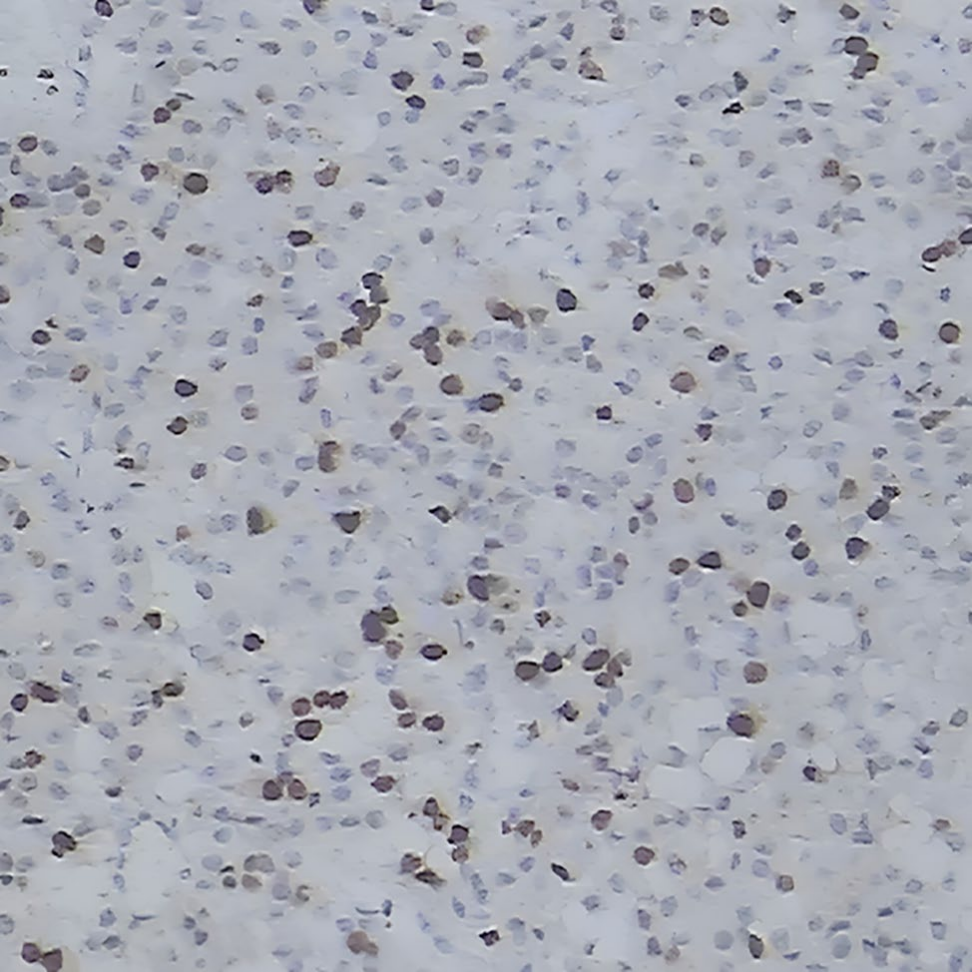
Ki-67-positive rate was about 40%

**Fig. 6:**
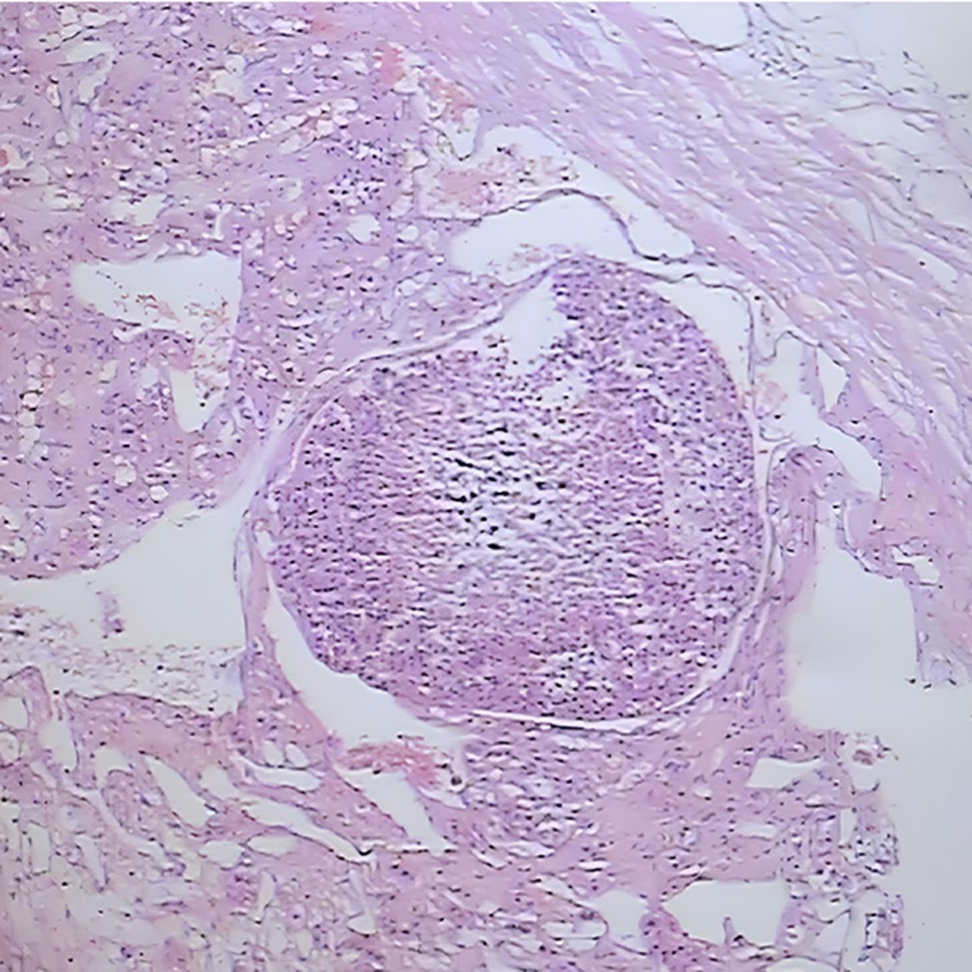
Intravascular cancer embolus

**Fig. 7:**
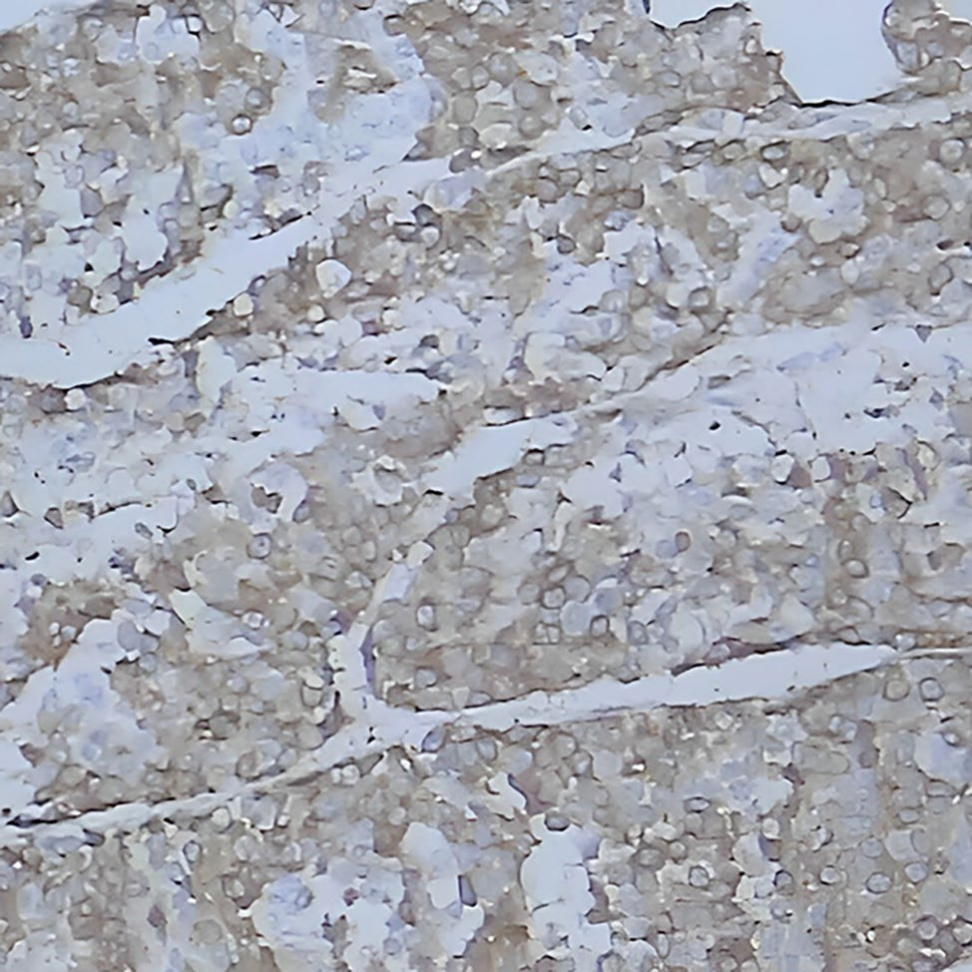
Inhibin positive

Two months after delivery, the patient underwent surgery of “excision of the right adrenal gland and space-occupying lesion + partial resection and repair of vena cava + stripping of right renal capsule” in our hospital. During the operation, a tumor about 10 cm in size was seen in the right retroperitoneum, with a fixed, position and hard texture, which was closely related to the right liver, kidney, and retrohepatic inferior vena cava. The tumor tissue was sent for pathological examination after the operation, and the results indicated an adrenal space-occupying grayish red nodular mass (10*7.5*7.5 cm), and the cut surface was grayish red and grayish yellow with medium texture, accompanied by hemorrhage and necrosis. A histopathologic description of cells is arranged in an acinar shape. (Fig. 4). Immunohistochemistry confirmed the diagnosis of right adrenocortical carcinoma, inhibin(+), Ki67(+, 40%), Vimentin(+) and demonstrated positive results for intravascular cancer embolus and capsule invasion (Fig. 5, Fig. 6 and Fig. 7).

Considering the patient’s condition, obstetrics and urology department recommended chemotherapy. After 3 months of follow-up, she has completed chemotherapy at another hospital. During the follow-up, she survived with no clinical evidence of disease recurrence.

## Discussions

ACC is a rare endocrine malignancy, and the etiology of most sporadic cases is unknown [4]. ACC typically involves the zona glomerulosa, zona fasciculata, or zona reticularis, and larger tumors can also cause adrenal medulla involvement [5]. The diagnosis of ACC is mainly based on clinical manifestations combined with endocrine examination, imaging examination, and pathological examination. Functional ACC is relatively easy to be diagnosed, manifested by increased cortisol, Cushing’s syndrome, hyperandrogenism, or primary aldosteronism clinically. However, non-functional ACC often develops insidiously and tends to trigger non-specific symptoms, such as anemia, weight loss, back pain, etc. Due to the presence of non-functional ACC, ACC cannot be excluded in patients with normal laboratory indicators. Pathological examination is the gold standard for the diagnosis of ACC [6].

Adrenal disorders are relatively rare in pregnancy, and in most cases, the diagnosis can only be confirmed at autopsy. The pathophysiologic repercussions of adrenal disorders are enormous to both mother and fetus, leading to significant maternal and fetal morbidity. Hence, timely diagnosis and appropriate treatment are critical to the prognosis of the mother and fetus [7–9].

Pregnancy-associated ACC is even less seen. The reported clinical symptoms of the vast majority of ACC patients in pregnancy are atypical, mainly including hypertension, diabetes, fatigue, depression, acne, etc. What is worse is that ACC in pregnancy is usually found at the advanced stage, resulting in a larger tumor than that observed in non-pregnant patients [10].

A retrospective study that compared 12 women diagnosed with ACC during pregnancy or immediately after delivery with non-pregnancy­diagnosed ACC patients, and found that patients diagnosed during pregnancy or postpartum had larger tumor volumes. However, the survival rate of patients diagnosed during pregnancy was 50% at one year and only 13% at five years [11]. Several studies clinical and in vitro tests that show a link between adrenal cortical proliferation and pregnancy [12]. Some researchers suggested that pregnancy is a risk factor for the deterioration of ACC [13]. Data on fetal survival in pregnancy with ACC are unknown, reported cases could be biased toward the publication of successful pregnancy outcomes.

It has been pointed out that more than 17% of tumor patients are accompanied by diabetes and abnormal elevation of blood glucose [14]. The impact of tumors on glucose metabolism is closely related to the energy metabolism characteristics of tumor cells, the secretion of ectopic hormones, and the destructive effect of tumor cells. On the one hand, the increase in blood glucose in tumor patients is related to the uptake of glucose and the production of lactate by tumor cells under aerobic conditions (Warburg effect). In addition, alternations in cell metabolism mediated by oncogenes can cause malignant cells to produce metabolic waste, promote the inflammatory response of immune cells in the tumor microenvironment, induce the transduction of the classic IL-6Rα/gp130/pSTAT3 pathway in the liver, facilitate the transcription of suppressor of cytokine signaling 3 (SOCS3), the target gene of *STAT3*, and destroy insulin receptor substrate-1 (IRS-1) and IRS-2, eventually resulting in insufficient glucose uptake and hyperglycemia [15]. On the other hand, pheochromocytoma, adrenocortical tumor, pituitary tumor, and glucagonoma can stimulate the secretion of hyperglycemic hormones that antagonize insulin, such as catecholamine hormones, adrenal cortical hormone, and glucagon.

Numerous studies have proven the functional alternations of the hypothalamic-pituitary-adrenal (HPA) axis in patients with type 2 diabetes, especially the high secretion of cortisol. Some scholars have proposed that there is a reciprocal causation relationship between hyperglycemia and high cortisol secretion. Glucocorticoids can not only promote glycogenolysis, inhibit liver glycogen synthesis, and stimulate gluconeogenesis, but also inhibit insulin secretion and thus lead to elevated blood glucose [16, 17]. Adrenal cortical carcinoma (ACC) is a rare primary neoplasm of the adrenal cortex, often accompanied by excessive cortisol secretion, which can increase the incidence of hyperglycemia. Blood glucose control can be significantly improved in patients undergoing adrenal adenoma resection surgery [18, 19].

According to reports, a woman affected by GDM was accidentally diagnosed with pancreatic cancer during the growth scan in the third trimester of pregnancy, so the growth scan in the third trimester of pregnancy is also very important in diagnosing the maternal abdominal mass [20].

## Conclusion

This study reported a rare case of ACC in pregnancy, that presented with gestational hypertension. Due to an accurate abdominal ultrasound evaluation the suspicion of an adrenal mass occurred.

Surgical treatment is the first choice for patients dur­ing an early second trimester of pregnancy who have an apparent mass on the adrenal gland, medication is usually ineffective. The best time for surgical intervention during pregnancy is the second trimester.In our opinion, after explaining the relative risks and surgical expertise involved in each case, individualized treatment should be carried out according to the preferences of patients and surgeons.

This study suggests that hypertensive diseases occurring in pregnancy should receive enough attention, and the possibility of adrenal diseases should be differentiated and excluded, so as to make a timely diagnosis and active treatment.

## Data Availability

Datasets used and/or analyzed in the current study are available from the corresponding author by request.

## Abbreviations

ACC: Adrenocortical carcinoma
OGTT: Oral glucose tolerance test
